# Extemporaneous dermatological compounding in hospital pharmacies, Northwest Ethiopia

**DOI:** 10.3389/fpubh.2025.1486936

**Published:** 2025-05-30

**Authors:** Ephrem Mebratu Dagnew, Ashenafi Kibret Sendekie, Wondale Tsega, Biset Asrade Mekonnen, Melese Getachew

**Affiliations:** ^1^Department of Pharmacy, College of Medicine and Health Sciences, Debre Markos University, Debre Markos, Ethiopia; ^2^Department of Clinical Pharmacy, School of Pharmacy, College of Medicine and Health Sciences, University of Gondar, Gondar, Ethiopia; ^3^Curtin Medical School, Faculty of Health Sciences, Curtin University, Bentley, WA, Australia; ^4^Department of Internal Medicine, School of Medicine, College of Medicine and Health Sciences, Debre Tabor University, Debre Tabor, Ethiopia; ^5^Department of Pharmacy, School of Health science, College of Medicine and Health sciences, Bahir Dar University, Bahir Dar, Ethiopia

**Keywords:** compounding, extemporaneous preparation, dermatological disease, skin preparations, Ethiopia

## Abstract

**Background:**

Dermatological problems are among the leading causes of hospital visits in Ethiopia. Extemporaneous compounded products are currently used by many patients with different disorders. The aim of the study was to evaluate the most commonly prescribed extemporaneously compounded products compounding practice and applicability of compounding guidelines at five randomly selected hospitals in Northwest Ethiopia.

**Methods:**

A hospital-based multicenter descriptive cross-sectional study was conducted by retrospectively analyzing prescription records for dermatological compounding from January to April 2023. A total of 423 prescriptions from hospital pharmacies were systematically selected. Data related to skin condition patterns, product selection, and dosage form types were extracted using a semi-structured data extraction tool and analyzed using SPSS version 25.0 at a significance level of 5%.

**Results:**

A total of 423 prescriptions containing dermatological products for compounding were analyzed. Most patients were female (82.1%) and aged 30–64 years (46.3%). Melasma (82.9%), acne vulgaris (68.6%), acute dermatitis (63.27%), and Rosacea (61.54%) were the four top dermatological diseases for which compounding preparations were prescribed. More than half of the prescriptions (56.26%) contained a combination of two drugs. Most compounded dosage form was semisolid preparations (95.98%), while the remaining 17 (4.02%) were liquids. Salicylic acid (35.39%) was the most frequently prescribed active ingredient, followed by Clobetasone (13.03%) and Betamethasone (10.01%). Vaseline (47.62%) and Nivea cream (44.3%) were the most commonly used excipients for compound preparations. Hydroquinone (4%) with Nivea cream (30gm) (17.0%), followed by salicylic acid (5%) + Betamethasone (75 g) + Vaseline (20 g) (10.6%) were the most commonly prescribed dermatological formulations.

**Conclusion:**

Dermatological disease is more prevalent in the study area. and extemporaneous compounding is a common element of pharmaceutical care. Extemporaneous and topical semisolid preparations containing two or more active ingredients are the most widely compounded and prescribed products. This study suggests the application of good manufacturing practices and componding guidelines for extemporaneous compounding of dermatological formulations to ensure efficacy, quality, and safety.

## Introduction

BOX 1 **Contributions of the literature.**

**Table:** 

There is limited evidence of the contributions of healthcare practice.
There is evidence of the applicability of compounding guidelines in Ethiopia.

Nearly one-third of the world’s population suffers from skin disorders, which rank as the fourth most common non-fatal disease burden worldwide (1), with an annual percent increase of 46.8% between 1990 and 2017 (2, 3). Skin conditions are among the most prevalent health issues in the world and have a significant financial and health impact. The psychological, social, and financial effects of skin disease on patients, their families, and society at large are all included in the multifaceted concept of the burden of skin disease. The two most prevalent skin conditions in poor nations are scabies and psoriasis (4, 5). In Ethiopia, dermatological issues account for 25% of cases in the Outpatient Department (OPD), making it one of the main reasons patients visit hospitals. Psychosocial consequences from skin diseases can have a major negative impact on patients’ quality of life (6).

Pharmaceutical compounding involves the combination, mixing, diluting, reconstructing, or alteration of a drug/bulk drug substance to create medications tailored to individual patient needs (7). It is an art or science to combine various chemical elements into a functional medicament. Extemporaneously compounded products are prepared specifically for an individual patient for immediate use and may include modifications to commercially manufactured products, such as the preparation of a suspension from tablets or the preparation of a product from individual raw materials (8).

Extemporaneous compounding is still a vital pharmaceutical service that continues to provide crucial patient populations with access to life-saving medications despite the development of commercial pharmaceutical manufacture and the widespread availability of contemporary medications (9, 10). One advantage of extemporaneously compounded products over commercial products is their flexibility for individual needs. Moreover, they have paramount importance in cases where individuals are sensitive to the ingredients of commercially available products, and even they may become the only way someone can receive a treatment that has been deemed insufficiently profitable by pharmaceutical companies (9–11).

Errors in extemporaneous compounding can occur at any point in the process, from prescribing medication to giving the patient their ready medication. Incorrect calculations and microbiological contamination are among the most serious errors, which may result in death. There were cases reported where the active component was absent from the final compounded preparation, where the active ingredient or excipients were wrong, or where the product was packaged incorrectly. It is crucial to note that unfavorable precipitation or nonhomogeneous mixing may arise from physicochemical problems, disregard for preparation guidelines, or improper preparation technology of extemporaneous medicines (12–14).

Ethiopian pharmacy practice, which included pharmaceutical compounding and manufacturing, was discovered at a very young age and was virtually forgotten in hospital settings (15). Recently, the Ministry of Health in Ethiopia has paid some attention to this service, which is regarded as one endeavor. Dermatological preparations are one of the services that a technical working group has been formed to help public hospitals provide this service (12, 15, 16).

Currently, many hospitals and community pharmacists in Ethiopia are increasingly incorporating the practice of extemporaneous compounding into their existing services (Box 1). However, data is lacking on the practice of prescribing, compounding, and use of extemporaneously compounded products in many hospitals. In addition, gaps have been observed in the facilities with the country’s regulatory requirements and their quality, efficacy, and safety (12, 16, 17). The safety, quality, and performance of compounded preparations depend on correct ingredients and calculations, accurate and precise measurements, and appropriate formulation conditions and procedures (12, 17).

Therefore, this study evaluated the existing practice of prescribing and compounding extemporaneous dermatological preparations, the applicability of extemporaneous compounding guidelines, and identified the most common dermatological diseases and the most commonly prescribed extemporaneous dermatological preparations at five randomly selected hospitals in northwest Ethiopia.

## Materials and methods

### Study setting and design

A retrospective multicenter descriptive cross-sectional study was conducted among patients with dermatological diseases who were followed at selected hospitals in Northwest Ethiopia. The study was conducted at five comprehensive and specialized hospitals in Northwest Ethiopia from January 1st to April 30, 2023. The hospitals were the University of Gondar Comprehensive and Specialized Hospital (UoGCSH), Felege Hiwot Comprehensive and Specialized Hospital (FHCSH), Tibebe Ghion Comprehensive and Specialized Hospital (TGCSH), Debre-Markos Comprehensive and Specialized Hospital (DMCSH), and Debre-Tabor Comprehensive and Specialized Hospital (DTCSH).

The UoGCSH is located in the west of the Central Gondar Administrative Zone, which is, 747 km from, Addis Ababa, the capital city of Ethiopia, in the Northwest direction. It is the capital city of the central Gondar Zone of the Amhara Region. It provides primary and referral healthcare services for nearly 5 million people.

FHCSH is also one of the largest referral hospitals in the Amhara region, located in Bahir-Dar, 565 km, northwest Ethiopia’s capital, Addis Ababa. It serves as a comprehensive hospital a population of seven million with approxamtely 1,200 outpatient consultations daily. Two specialized dermatology clinics accept referred cases from district hospitals in the region and neighboring areas.

TGCSH is also one of the largest teaching hospitals situated in Bahir-Dar. It provides primary and referral healthcare services to more than 5.5 million people living in Bahir-Dar town and neighborhood woreda and zones.

DMCSH is the largest hospital in East Gojjam and is located 295 km northwest of Addis Ababa, the capital of Ethiopia. It provides primary and referral healthcare services to nearly 5 million people in its catchment area.

Similarly, DTCSH is 295 km from Ethiopia’s capital, Addis Ababa, and 265 km from Bahir Dar, the capital of the Amhara regional state. It provides primary and referral healthcare services for nearly 5 million people.

### Sample size determination and sampling techniques

The single population proportion formula was used to calculate the required sample size by considering the following assumptions: 50% population distribution, 95% confidence level, and W = 5% margin of error (absolute level of precision).


$$n=\frac{z^{2}*p*(1-p)}{w^{2}}$$

Where *n* is the minimum sample size, Z is the reliability coefficient for the desired interval (CI) for 95% = 1.96, and *p* is the prevalence of the prescribing pattern of dermatological compounding. After considering 10% contingency for a possible incomplete response, the final sample size was 423.

A systematic random sampling technique was used to select patients prescriptions. Initial samples were selected using simple random sampling using the lottery method. Then, eligible prescriptions were included in the study using a sampling frame until the final sample size was maintained.

### Eligibility criteria

All extemporaneous compounding prescriptions in the compounding pharmacy at selected Northwest hospitals were included in the study. Prescriptions with incomplete items, e.g., without a drug item, i.e., those that contain only bases, were excluded from the study.

### Data collection instruments, procedures, and quality control measures

The data were collected using a semi-structured data extraction tool prepared after reviewing earlier studies. The specific types of data necessary to assess the patterns of extemporaneous preparations and dermatological problems were recorded for each patient prescription.

To ensure the quality of the data, the data collectors were trained for two days by the principal investigator. The data collection process was conducted under close supervision by the investigators, and the collected data were checked daily for completeness during the data collection period. The data collection tool was also pretested on 21 patients (5% of the calculated sample size) from the recorded prescriptions of the dermatology clinic to check the acceptability and consistency of the data collection tool 2 weeks before the actual data collection. In addition, experts in the area of face validity and approval reviewed the questionnaire.

### Data entry and analysis

After collection of the data, the data were entered and analyzed using SPSS version 25. A descriptive analysis was performed to explore the patterns of extemporaneous preparations and common dermatological problems implicated in the prescription of these preparations. Frequencies and proportions were used to describe sociodemographic characteristics of the patients.

## Results

### Patterns of participants’ dermatological disorders

Analyses were performed on 423 prescriptions that contained dermatological products for compounding. Most patients (46.3%) were female (82.1%) and between the ages of 30–64. The four top skin conditions for which compounded remedies were prescribed were Melasoma (81.9%), Acne vulgaris (68.6%), Acute dermatitis (63.27%), and Rosacea (61.54%). Among the 423 reported skin diseases, melasma was found to predominantly affect female patients (82.9%) in the age group of 30–64 years (46.7%). The age range of 18–29 years was the main demographic impacted by acne vulgaris, which was the second most prevalent dermatological issue among females (68.6%). Table 1 presents the thorough diagnosis and information on patient education.

**Table 1:** Distribution of the top ten dermatological conditions for which compounded medications were prescribed by age and sex at selected hospitals in Northwest Ethiopia, 2023 (*N* = 423).

Diagnosis	Sex		Age (yr)					Total
	Male	Female	<5	5–17	18–29	30–64	≥65	
Melasma	12 (17.91)	55 (82.09)	0	6 (8.96)	30 (44.78)	31 (46.27)	0	67
Acne vulgaris	16 (31.37)	35 (68.63)	0	15 (29.41)	32 (62.75)	4 (7.84)	0	51
Acute dermatitis	18 (36.73)	31 (63.27)	0	19 (38.78)	15 (30.61)	9 (18.37)	6 (12.24)	49
Rosaceae	15 (38.46)	24 (61.54)	0	0	15 (38.46)	24 (61.54)	0	39
Lichen simplex chronicus (LSC)	6 (16.22)	31 (83.78)	0	0	18 (48.65)	19 (51.35)	0	37
Seborrheic Dermatitis (SD)	3 (13.04)	20 (86.96)	0	10 (43.48)	3 (13.04)	10 (43.48)	0	23
Wart	16 (84.21)	3 (15.79)	0	3 (15.79)	16 (84.21)	0	0	19
Eczema	13 (68.42)	6 (31.58)	0	3 (15.79)	4 (21.05)	9 (47.37)	3 (15.79)	19
Psoriasis	6 (40)	9 (60)	3 (20)	3 (20)	9 (60)	0	0	15
Tania pedis	3 (25)	9 (75)	0	0	3 (25)	9 (75)	0	12
Lichen planus	0	12 (100)	0	0	6 (50)	6 (50)	0	12
Perioral dermatitis	0	10 (100)	0	0	0	10 (100)	0	10
SD + Rosaceae	3 (33.33)	6 (66.67)	0	0	0	9 (100)	0	9
Atopic dermatitis (AD)	0	8 (100)	0	4 (50)	0	4 (50)	0	8
Scabies	4 (57.14)	3 (42.86)	4 (57.14)	0	3 (42.86)	0	0	7
Keratosis pilaris	6 (100)	0	0	0	0	0	6 (100)	6
Pityriasis Rubra Pilaris	0	6 (100)	3 (50)	0	3 (50)	0	0	6
Keratosis pilaris	4 (100)	0	0	4 (100)	0	0	0	4
Genital wart	0	4 (100)	0	0	4 (100)	0	0	4
AD + Eczema	4 (100)	0	0	0	0	4 (100)		4
PPK + Acroid	4 (100)	0	0	0	4 (100)	0	0	4
Xerosis	3 (100)	0	0	0	0	3 (100)	0	3
Folliculitis	3 (100)	0	0	0	0	3 (100)	0	3
Ulcer	3 (100)	0	0	0	0	3 (100)	0	3
Keratoderma	3 (100)	0	0	0	0	3 (100)	0	3
Planter wart	3 (100)	0	0	3 (100)	0	0	0	3
Corn	0	3 (100)	0	0	3 (100)	0	0	3

LSC: Linchus symplex chronics, SD: Seborrheic Dermatitis, AD: Atopic Dermatitis, PPK: Palmoplantar keratoderma.

### Number of active ingredients used in extemporaneous dermatological preparations

A total of 13 Active pharmaceutical Ingredients (API) were prescribed to be developed as extemporaneous dermatological preparations. Majority of the prescriptions, 238 (56.26%) contained two drugs (Table 2).

**Table 2:** Commonly prescribed combination of active ingredients for dermatological preparation at selected hospitals in Northwest Ethiopia, 2023 (*N* = 423).

Formulation	Frequency	Percentage
Salicylic acid + clobetasone	65	15.37
Salicylic acid + betametasone	61	14.42
Salicylic acid + sulfur	42	9.93
Urea + clobetasone	26	6.15
Salicylic acid + mometasone + sulfur	19	4.49
Salicylic acid + fluconazole	16	3.78
Urea + betamethasone	15	3.55
Salicylic acid + mometasone	10	2.36
Salicylic acid + ketoconazole	3	0.71

### Dosage forms used in extemporaneous dermatological preparations

Of the total 423 formulations prepared extemporaneously, 406 (95.98%) were semisolid preparations, while the remaining 17 (4.02%) were liquids (Table 3).

**Table 3:** Type of dosage forms used for extemporaneous dermatological preparation at selected hospitals in Northwest Ethiopia, 2023 (*N* = 423).

Type of dosage forms	Frequency	Percentage
Semisolid preparations	406	95.98
Liquid preparations	17	4.02
Total	423	100.00

### Active ingredients used in extemporaneous dermatological preparations

The two extemporaneous medications that were prescribed the most commonly were Salicylic acid (35.39%) and Clobetasone (13.03%), respectively (Table 4).

**Table 4:** Number of APIs per encounter of dermatological preparations at selected hospitals in Northwest Ethiopia, 2023 (*N* = 423).

API	Frequency	Percentage
Salicylic acid	258	35.39
Clobethasone	95	13.03
Betamethasone	73	10.01
Hydroquinone	72	9.88
Sulfur	65	8.92
Urea	57	7.82
Metronidazole	37	5.08
Momethasone	33	4.53
Fluconazole	19	2.61
KOH	10	1.37
TCA	4	0.55
Ketoconazole	3	0.41
AlCl_3_	3	0.41

### Excipients used in extemporaneous dermatological preparations

**Figure 1:**
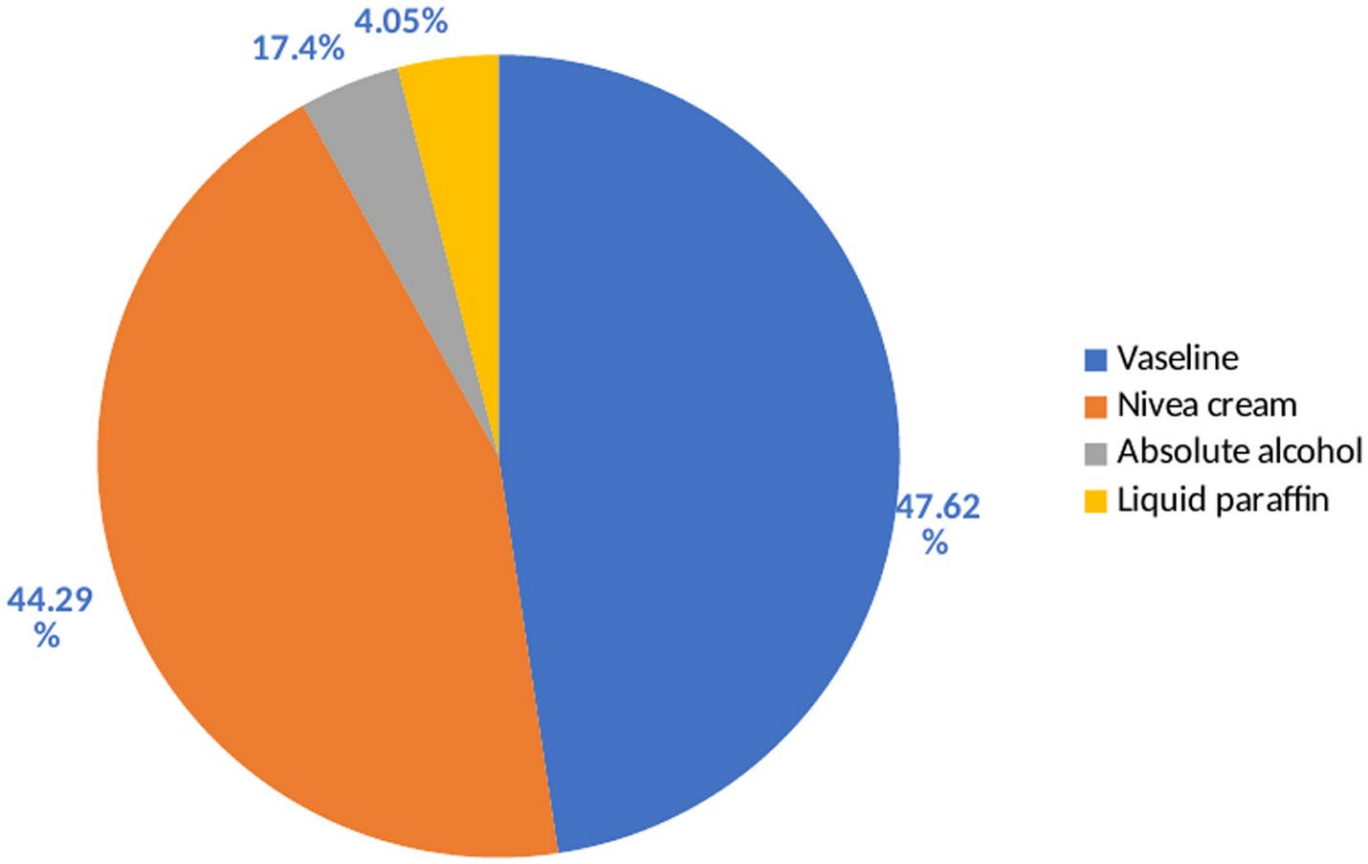
Excipients used for the compounding of dermatological preparations at selected hospitals in Northwest Ethiopia between January and April 2023 (*N* = 423).

Among the frequently utilized excipients for the compounding of dermatological preparations, Vaseline 200 (47.6%), Nivea cream 186 (44.3%), followed by absolute alcohol and liquid paraffin 17 (4.05%), respectively (Figure 1).

### Characteristics and composition the active ingredients most frequently used in dermatological preparations

Hydroquinone (4%) with Nivea cream (30gm) (17.0%), followed by salicylic acid (5%) + Betamethasone (75 g) + Vaseline (20 g) (10.6%) were most commonly prescribed dermatological formulations in the study area (Table 5).

**Table 5:** Compositions of formulations prescribed for dermatological preparation at selected hospitals in Northwest Ethiopia, 2023 (*N* = 423).

Regimen	Frequency	Percentage
Hydroquinone (4%) + Nivea cream (30 g)	72	17.0
Salicylic acid (5%) + Betametasone (75 g) + Vaseline (20 g)	45	10.6
Metronidazole (1%) + Nivea cream (30 g)	37	8.7
Salicylic acid (2%) + Sulfur (3%) + Nivea cream (30 g)	33	7.8
Salicylic acid (6%) + Clobetasone (45 g) + Vaseline (60 g)	30	7.1
Salicylic acid (2%) + Sulfur (3%) + Nivea cream (30 g) + mometasone (30 g)	19	4.5
Urea (4%) + Betametasone (60 g) + Vaseline (100 g)	12	2.8
Salicylic acid (2%) + Clobetasone (30 g) + Vaseline (30 g)	12	2.8
Urea (15%) + Clobetasone (30 g) + Vaseline (50 g)	12	2.8
Salicylic acid (4%) + Clobetasone (30 g) + Vaseline (50 g)	11	2.6
Urea (3%) + Clobetasone (30 g) + Vaseline (50 g)	11	2.6
KOH (10%) + Absolute alcohol (50 mL)	10	2.4
Salicylic acid (3%) + fluconazole (50 g) + Vaseline (50 g)	9	2.1
Salicylic acid (2%) + Sulfur (5%) + Nivea cream (30 g)	9	2.1
Salicylic acid (4%) + Betametasone (3%) + Nivea cream (30 g)	9	2.1
Salicylic acid (3%) + mometasone (45 g) + Liquid paraffin (90 mL)	7	1.7
Urea (15%g) + Vaseline (50 g)	7	1.7
Salicylic acid (7%) + Vaseline (20 g)	6	1.4
Salicylic acid (4%) + Betametasone (30 g) + Vaseline (50 g)	4	0.9
Sulfur (5%) + Nivea cream (30 g)	4	0.9
mometasone (45 g) + Liquid paraffin (90 mL)	4	0.9
Clobetasone (45 g) + Vaseline (60 g)	4	0.9
TCA (70%) + Absolute alcohol (100 mL)	4	0.9
Salicylic acid (4%) + fluconazole (100 mg) + Vaseline (100 g)	4	0.9
Salicylic acid (4%) + Clobetasone (30 g) + Vaseline (30 g)	3	0.7
Salicylic acid (3%) + fluconazole (50 g) + Vaseline (60 g)	3	0.7
Urea (10%) + Vaseline (50 g)	3	0.7
Urea (20 g) + Vaseline (20 g)	3	0.7
Salicylic acid (3%) + mometasone (40 g) + Vaseline (40 g)	3	0.7
Urea (5%) + Clobetasone (50 g) + Vaseline (20 g)	3	0.7
Urea (8%g) + Vaseline (50 g)	3	0.7
Urea (10%g) + fluconazole (45 mg) + Vaseline (50 g)	3	0.7
Salicylic acid (3%) + Betametasone (30 g)	3	0.7
Salicylic acid (8%) + Vaseline (30 g)	3	0.7
Salicylic acid (5%) + Clobetasone (30 g) + Vaseline (50 g)	3	0.7
Salicylic acid (6%) + Ketoconazole (2%) + Vaseline (30 g)	3	0.7
AlCl3 (20%) + Absolute alcohol (20 mL)	3	0.7
Salicylic acid (5%) + Clobetasone (45 g) + Liquid paraffin (50 mL)	3	0.7
Salicylic acid (2%) + Clobetasone (30 g) + Liquid paraffin (90 mL)	3	0.7
Salicylic acid (3%) + Nivea cream (30 g)	3	0.7

## Discussion

Most patients in this study were female, and females were more likely to suffer from autoimmune and allergy diseases as well as skin conditions such as psychosomatic issues, pigmentary disorders, specific hair diseases, and psychosomatic problems. Although differences in skin structure and physiology due to the impact of sex hormones may influence gender disparities in skin diseases, the processes behind these differences remain largely unclear (18).

Melasma was found to predominantly affect female patients (82.9%) in the age group of 30–64 years (46.7%), this result is similar to previous studies as epidemiological review showed a clear female predominance was observed in the reports of the disease, ranging from 9 or 10 to 1 (19). This could be because female sex hormones are well-known risk factors for the development of melasma, and their preponderance in fertile women reinforces this hypothesis (20). In contrast to earlier research among Nigerian undergraduates, where there was a substantial difference between the severity of acne in males and females, acne vulgaris was the second most prevalent dermatological issue among females (68.6%), and mostly affected the age period of 18–29 years (68.6%) (21, 22). The female predominance in this study could be because stress is a significant factor in acne and females are more likely than males to suffer from stress-related depressive disorders and anxiety (23).

For most skin conditions in this study, extemporaneous dermatological products were prescribed for patients above the age of 18 years, and this result is similar to that of a previous study. For majority of skin problems, compounded dermatological products were meant for adults and older adult patients. The possible reason is dosage modifications or combining commercial products, which is considered in the study’s finding that the majority (56.3%) of prescriptions carry the combined drugs (5). This finding is opposite to other studies in which children under 12 years of age received relatively more extemporaneous compounded dermatological medicines than other age groups, which could be due to the need for the patient to be fashioned or fitted to resemble a tailor’s therapy for children (24–27).

The majority (60.76%) of compounded prescriptions carry two or more drugs, which is supported by other similar studies in ALERT hospital, were the majority of prescriptions (65%) carry more than one active pharmaceutical substance (5). However, the rate was greater than in our study in the compounded prescription pattern study carried out in Jogjakarta Province hospital (28) because of the nature of some skin disorders that could respond to a single active medicinal ingredient. A survey of Australian general practitioners mentioned these as the major reasons for prescribing certain extemporaneous formulations (29).

Among prescribed drugs, salicylic acid (35.39%) and clobetasone (13.03%) were the most commonly used active pharmaceutical substances in the hospital for extemporaneous compounded products, which is similar to a previous study in which salicylic acid (38.0%) was the most commonly used ingredients (2). Therefore, their continued availability is necssary to satisfy the specific needs of patients. In contrast to clobetasone, which was purchased as a cream from the market and used for compounding, salicylic acid is available in the hospital in powder form.

Ointments and creams comprised the majority of dose forms from the compounded products in the study area, which is consistent with other similar studies (26, 30, 31). Dermatological treatments offered in the form of these dosage forms may be due to the type of skin lesions (dry or leaking moisture) found on patients and the ease of accessibility to vehicle agents.

Several semisolid formulations are available for topical applications intended to address the type and location of skin problems. Ointments are substances used on the skin to soothe or heal wounds, burns, rashes, scrapes, and other skin problems. Also called unguent. The possible reason for the preference of these semisolid dosage forms is their ease of application, rapid formulation, and ability to topically deliver a wide variety of drug molecules compared with the application site in liquid preparations (18, 23).

### Strength and limitations

A strength of the current study is its multicenter nature, which increases the generalizability of the findings. Despite the findings of this study, we acknowledge that it has limitations. Drugs that were not available in the hospital are excluded and extemporaneous products from community pharmacies or outside the hospital were not included.

## Conclusion

Dermatological disease is more prevalent in the study area. Females are mostly affected. The four top prevalent dermatological diseases are: melasma, acne vulgaris, acute dermatitis, and rosacea. Extemporaneous topical semisolid preparations containing two or more active ingredients are the most widely compounded and prescribed products for pharmaceutical care. Salicylic acid, clobetasone, and betametasone are widely used active pharmaceutical ingredients. Vaseline and Nivea cream are the most commonly used vehicles for compounding preparations. Hydroquinone (4%) with Nivea cream (30gm), Salicylic acid (5%) + Betametasone (75 g) + Vaseline (20 g) were the most commonly prescribed dermatological formulations. There are concerns related to the applicability of a country regulatory requirements and good manufacturing practices, and their efficacy, quality, and safety. So, this study suggests that application of good manufacturing practices and compounding guidelines for extemporaneous compounding of dermatological formulations to ensure efficacy, quality, and safety.

## Data Availability

The original contributions presented in the study are included in the article/supplementary material, further inquiries can be directed to the corresponding author.
