# Correction: Extemporaneous dermatological compounding in hospital pharmacies, Northwest Ethiopia

**DOI:** 10.3389/fpubh.2025.1685055

**Published:** 2025-09-05

**Authors:** Ephrem Mebratu Dagnew, Ashenafi Kibret Sendekie, Wondale Tsega, Biset Asrade Mekonnen, Melese Getachew

**Affiliations:** ^1^Department of Pharmacy, College of Medicine and Health Sciences, Debre Markos University, Debre Markos, Ethiopia; ^2^Department of Clinical Pharmacy, School of Pharmacy, College of Medicine and Health Sciences, University of Gondar, Gondar, Ethiopia; ^3^Curtin Medical School, Faculty of Health Sciences, Curtin University, Bentley, WA, Australia; ^4^Department of Internal Medicine, School of Medicine, College of Medicine and Health Sciences, Debre Tabor University, Debre Tabor, Ethiopia; ^5^Department of Pharmacy, School of Health science, College of Medicine and Health sciences, Bahir Dar University, Bahir Dar, Ethiopia

**Keywords:** compounding, extemporaneous preparation, dermatological disease, skin preparations, Ethiopia

Author Ashenafi Kibret Sendekie was erroneously assigned to affiliation “Department of Internal Medicine, School of Medicine, College of Medicine and Health Sciences, Debre Tabor University, Debre Tabor, Ethiopia”. This affiliation has now been removed for author Ashenafi Kibret Sendekie.

There was a mistake in the caption of Figure 1 as published. This caption “Number of DRPS among patients diagnosed and treated with cancer in UoG and felegehiwot comprehensive and specialized hospitals Northwest, Ethiopia, April 1 to July 15, 2021.” was an error and did not relate to the current figure. The corrected caption of Figure 1 appears below.

“Excipients used for the compounding of dermatological preparations at selected hospitals in Northwest Ethiopia between January and April 2023 (*N* = 423).”

The original version of this article has been updated.

